# Antioxidant Characterization of Six Tomato Cultivars and Derived Products Destined for Human Consumption

**DOI:** 10.3390/antiox12030761

**Published:** 2023-03-21

**Authors:** Anna Rita Bianchi, Ermenegilda Vitale, Valeria Guerretti, Giancarlo Palumbo, Isabella Maria De Clemente, Luca Vitale, Carmen Arena, Anna De Maio

**Affiliations:** 1 Department of Biology, University of Naples Federico II, Via Cinthia, 80126 Napoli, Italy; 2 Department of Economy, Management, Institutions, University of Naples Federico II, Via Cinthia, 80126 Napoli, Italy; 3 Institute for Agricultural and Forestry Systems in the Mediterranean, National Research Council of Italy, P. le Enrico Fermi 1, Loc. Porto del Granatello, 80055 Portici, Italy; 4 NBFC—National Biodiversity Future Center, 90133 Palermo, Italy; 5 Istituto Nazionale Biostrutture e Biosistemi (INBB)—Consorzio Interuniversitario, Viale delle Medaglie d’Oro, 00136 Rome, Italy

**Keywords:** tomato, peel, seed, pulp, tomato puree, total water-soluble and fat-soluble antioxidant capacity, total phenolic content, ascorbic acid, lycopene

## Abstract

The consumption of fresh tomatoes and processed tomato products is widespread in the Mediterranean diet. This fruit is a valuable source of antioxidants and plays an important role in preventing oxidative stress. This study aimed to investigate the content of antioxidants and measure the total antioxidant capacity (ABTS and DPPH assays) in the peel, pulp, and seed fractions of six tomato cultivars. Finally, some bioactive compounds and total antioxidant activity were also determined in homemade tomato purees, since such homemade production is commonplace in Southern Italy. The level of antioxidants and total antioxidant capacity in each fraction were also calculated based on their actual fresh weight in the whole tomato. The overall results indicated that the peel and seeds of all analysed tomato cultivars contribute significantly to the antioxidant charge of the fruits. Consequently, consuming tomatoes without peel and seeds results in a substantial loss of compounds beneficial for human health. Our results also showed that phenolic and lycopene content, as well as antioxidant activities in all purees are higher than in fresh tomatoes. Based on this evidence, producing homemade tomato puree is a good practice, and its consumption helps prevent oxidative stress damage.

## 1. Introduction

The tomato berry is a versatile fruit that is eaten both fresh and as processed products. It is a staple ingredient of the Mediterranean diet, mainly because of its beneficial properties. It provides good levels of dietary fibre and varying amounts of all essential minerals and vitamins [1,2,3].

The nutritional content of tomato berries depends on biotic and abiotic factors. In addition, the physiological state of the plant, the moisture and salinity level of the substrate, the light quality and intensity, the ripening stage, temperature, presence of heavy metals, cultivar type, post-harvest conditions, and processing and storage conditions significantly influence the biosynthesis and concentrations of the substances present in the tomato [4].

The elevated consumption of fresh and processed tomatoes confers on this fruit the role of the primary source of antioxidant molecules (ascorbic acid, vitamin E, carotenoids, flavonoids, phenolic acids) involved in preventing a wide range of diseases [5].

The vitamin most commonly found in tomato berries is vitamin C, which benefits the immune system and promotes the absorption of iron and calcium [6,7]. In addition, tomato berries also contain: considerable amounts of vitamin K, which is necessary for proper coagulation [8]; vitamin A, which is essential for the immune system as well as cell regeneration and healthy skin [9]; vitamin E, which protects polyunsaturated membrane lipids from free radical attack and promotes enhanced humoral and cellular immune responses [10]; and finally, all the B-complex vitamins, which are essential for normal appetite, good vision, healthy skin, the nervous system, and red blood cell formation [11].

Among all the carotenoids in ripe tomatoes, lycopene is the most abundant [12] and is responsible for the red colour of this fruit. However, although tomatoes are a significant source of dietary lycopene, most of this compound contained in fresh tomatoes is present as trans isomers, whose bioavailability is very low [13].

Lycopene bioavailability depends on many factors, such as cis–trans isomerization and tomato processing [14]. For example, temperature and processing time increase the isomerization of lycopene to the cis isomer in processed tomato products. As a result, these products have higher lycopene bioavailability: thanks to the cis isomer’s shorter chain length, it is more soluble and more easily absorbed by human intestinal cells [13,15].

Tomato processing methods also increase lycopene bioavailability as they weaken the binding forces between lycopene and the tissue matrix [12,13]. Interestingly, lycopene has been widely shown to protect against a wide range of diseases, such as obesity and diabetes [16], Alzheimer’s disease [17], and several types of cancer [18].

Epidemiological evidence suggests that tomato is a potential factor in reducing serum levels of oxidative stress biomarkers. In support of lycopene’s role in preventing oxidative stress-related diseases, it has been demonstrated that daily consumption of 160 g of tomato sauce rich in lycopene produces a decrease in oxidized LDL cholesterol levels [19]. Lycopene reduces the risk of cardiovascular disease [20,21], breast cancer in postmenopausal women [22], ovarian cancer in premenopausal and postmenopausal women [23], and prostate cancer in men [24].

Lycopene seems to also play a skin protective role after exposure to UV irradiation [25], and higher serum levels of lycopene have been associated with reduced mortality in individuals with metabolic syndrome [26].

Toor and Savage [27] have compared the main antioxidants and total antioxidant activity in the peel, pulp, and seed fractions of three commercially grown New Zealand tomato cultivars. They have demonstrated that the peel of tomatoes contains significantly higher concentrations of phenols, flavonoids, lycopene, ascorbic acid, and antioxidant activity than the pulp and seeds.

It has also been reported that processed tomato products’ antioxidant capacity and antioxidant content, as well as their resulting health value, directly depend on industrial processing techniques (cold cracking, evaporation, pasteurization, etc.) [28,29]. To date, several studies have been conducted to examine the impact of processing techniques on the content of carotenoids and their isomerization [30], while few data are available on the content of ascorbic acid, total phenols, and tocopherols [29,31,32]. In any case, results are often conflicting because the content of bioactive compounds depends on the different processing techniques and conditions, and their processing sensitivity and stability also depend on the cultivar [5,33,34,35].

Based on what has been reported so far, the present work aimed to determine hydrophilic and lipophilic phenols, lycopene, and ascorbic acid content, and the total water-soluble and fat-soluble antioxidant capacity in six tomato landraces (Cherry tomato, “*Ciliegino*”; Smooth round tomato, “*Pomodoro tondo liscio*”; Round tomato sauce, “*Pomodoro tondo da sugo*”; “Datterino” tomato; “S. Marzano” tomato; and “Piccadilly” tomato). In detail, the antioxidant content and activities were determined in the peel, seed, and pulp fractions of each cultivar to assess whether removing the peel and the seeds may cause a significant loss of measured antioxidants.

The concentration of antioxidants and total antioxidant capacity level in each fraction was measured based on their actual fresh weight in the whole tomato. In addition, hydrophilic and lipophilic phenols, ascorbic acid, and lycopene concentrations and antioxidant capacities were also evaluated in homemade tomato purees.

## 2. Materials and Methods

### 2.1. Sample Preparation

*Tomato fruit.* Six varieties of healthy, ripe tomatoes (Cherry tomato, CT “*Ciliegino*”; Smooth round tomato, ST “*Pomodoro tondo liscio*”; Round tomato sauce, RTS “*Pomodoro tondo da sugo*”; “Datterino” tomato, DT; “S. Marzano” tomato, SMT; “Piccadilly” tomato, PT) were purchased from a supermarket near Naples. Each tomato’s seeds, pulp, and peel were carefully separated with a sharp knife. The peel was the outer epidermis; the seed fraction of the tomatoes consisted of the seeds and the jelly portion; the pulp consisted of the portion of the tomato that remained after the peel and seed fractions were removed. The fresh weight of the whole fruit, seeds, pulp, and peel were reported in Table 1. All fractions and whole tomatoes were stored at −20 °C until analysis.

*Tomato puree.* Tomato puree was obtained by pressing fresh, ripe whole tomatoes (2 kg). After washing them for 5 min with water and blanching at 85–100 °C for 3 min, each of the six tomato varieties was refined through a tomato press to obtain a traditional puree without seeds and peel. Glass jars (500 g) were filled with the tomato puree and then sealed. The filled jars were heated at 100 °C for 40 min in water and then cooled. The tomato purees were subjected to subsequent analysis after several days of storage.

### 2.2. Extraction of Water-Soluble and Fat-Soluble Antioxidants

The extraction of water-soluble and fat-soluble antioxidants from each cultivar’s peel, pulp, and seed fractions and tomato purees was conducted as described in Arena et al. [36], with some modifications. Hydrophilic extracts were obtained using absolute ethanol, while absolute acetone was used to extract fat-soluble antioxidants. All described procedures were performed on ice and in the dark. First, the whole samples were homogenized with a Polytron Ultra Turrax T8 (IKA-WERKE) and then 0.3 g of each homogenate was resuspended in 1.5 mL absolute ethanol. After shaking in the dark for 16–18 h, the samples were centrifuged at 8500 rpm for 30 min at 4 °C using an Eppendorf 5417 R centrifuge (Bio-Rad, rotor F 45-30-11). The first supernatant (hydrophilic extract) was transferred into new tubes; the pellet was extracted as previously described and the second supernatants were added to the first.

Finally, 1.5 mL of absolute acetone was added to the precipitates to obtain the fat-soluble extracts. The following experimental procedures are the same as described above.

### 2.3. Water-Soluble and Fat-Soluble Antioxidant Capacity

The free radical scavenging capacity of the water-soluble and fat-soluble extracts of fresh tomato fractions and purees was determined using the 2,2′ azino-bis (3-ethylbenzthiazoline-6-sulphonic acid) (ABTS^•+^) radical cation decolourization assay as described by Ariano et al. [37]. The ABTS^•+^ radical cation was generated by a reaction between 2.45 mM and 7 mM ABTS, in the dark, for 16 h at room temperature. The reaction mixture was diluted with ethanol to obtain an absorbance of 0.800 ± 0.050 at 734 nm and was utilized within two days. A volume of 15 µL of extracts were mixed with 1 mL of diluted ABTS^•+^ solution and then incubated at room temperature for 10 min. The decolouration resulting from cation reduction by antioxidants in the sample was measured at 734 nm using an LLG uniSPEC 2 UV/VIS-Spectrometer (Labware). Assays were performed with three dilutions of each extract, in duplicate. Trolox (6 hydroxy-2,5,7,8-trimethyl-chroman-2-carboxylic acid) (0–15 µM) was used to plot the standard curve. Antioxidant capacity was expressed as micromolar Trolox equivalents (TEAC) per 100 g fresh weight (FW).

Water-soluble and fat-soluble antioxidant capacity of whole fresh tomatoes and purees were also determined by DPPH assay [37]. A solution of 60 µM of DPPH^•^ in ethanol was prepared daily in the dark. Then, 50 µL of the extract was mixed with 1.95 mL of DPPH^•^ solution and incubated for 15 min. The decrease in absorbance at 517 nm was recorded using a spectrophotometer (uniSPEC 2 UV/VIS, Lab Logistics Group GmbH Labware, Germany). A standard curve was prepared by measuring the scavenging activities of the DPPH^•^ solution at different concentrations of Trolox (6.25, 12.5, 18.8, and 25 µM). The results were expressed as µM Trolox/100 g fresh weight.

### 2.4. Total Phenolic Content

Total phenolic content was determined in both hydrophilic and lipophilic extracts of fresh tomato fractions and purees by the Folin-Ciocalteu assay [38]. Briefly, 0.1 mL of hydrophilic extract was mixed with 2.5 mL of 10-fold diluted Folin-Ciocalteau reagent, and the reaction was neutralized by adding 2.0 mL of 7.5% (*w*/*v*) sodium carbonate (Na_2_CO_3_). After incubation for 2 h at room temperature, the absorbance of the reaction mixtures was measured at 760 nm spectrophotometrically (uniSPEC 2 UV/VIS-Spectrometer, Lab Logistics Group GmbH Labware, Meckenheim, Germany). Gallic acid was used as a standard, and the total phenolic content of the hydrophilic extracts was expressed in milligram gallic acid equivalents (mg GAE) per 100 g fresh weight (FW).

### 2.5. Lycopene

Lycopene concentration was extracted in the dark from 0.3 g of seeds, pulp, peel, and tomato purees with a mixture of 15 mL hexane:acetone:ethanol (2:1:1), as described in Periago et al. [39], with slight modifications. The total lycopene content was measured at 472 nm spectrophotometrically. Lycopene was used to prepare the standard curve, and the results were expressed as milligrams per 100 g fresh weight (FW).

### 2.6. Ascorbic Acid

The ascorbic acid (AsA) in the peel, pulp, and seed fractions and tomato purees of each cultivar was measured using the Ascorbic Acid Assay Kit (MAK074, Sigma-Aldrich, St. Louis, MO, USA), following the procedure reported by Costanzo et al. [40]. Briefly, 10 mg of sample was homogenized in 4 volumes of cold AsA buffer and then centrifuged at 13,000 rpm for 10 min at 4 °C to remove insoluble material. Next, the supernatant was mixed with AsA assay buffer to a final volume of 120 μL. The assay reaction was performed by adding the kit reagents to the samples. The ascorbic acid was determined by a coupled enzyme reaction, which developed a coloured (570 nm) product proportional to the amount of ascorbic acid in the sample. A standard calibration curve was used to quantify the ascorbic acid content, and the results were expressed as milligrams per 100 g fresh weight (FW).

### 2.7. Statistical Analysis

Statistically significant differences were assessed by one-way analysis of variance (ANOVA), followed by Holm-Sidak’s multiple comparisons test using the GraphPad Prism 8 Software. The results of total water-soluble and fat-soluble antioxidant capacities, as well as the phenolic, lycopene, and ascorbic acid contents, were reported as the mean ± standard deviation (SD), and the minimum level of acceptable significance was *p* < 0.05. The different letters in the figures indicate the significant differences (*p* < 0.001) observed when comparing the values of each fraction belonging to one variety with those measured in the others. The tables show the *p*-values (*p* < 0.05; *p* < 0.01; *p* < 0.001) obtained by multiple comparisons between the three fractions (peel vs. seeds, peel vs. pulp, and seeds vs. pulp).

## 3. Results

### 3.1. Antioxidant Capacity and Content in the Seeds, Pulp, and Peel Fractions of Different Cultivars

In all the cultivars, the highest levels of water-soluble and fat-soluble activities were found in the peel compared to both seeds and pulp. Moreover, the seeds showed higher values than the pulp.

**Figure 1:**
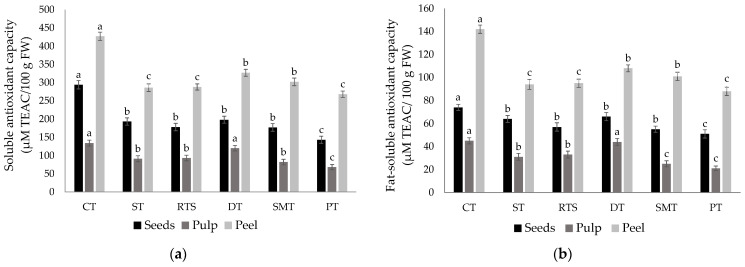
Water-soluble (**a**) and fat-soluble (**b**) antioxidant activity in the seeds, pulp, and peel fractions of each tomato cultivar: Cherry tomato (CT), Smooth round tomato (ST), Round tomato sauce (RTS), Datterino tomato (DT), S. Marzano tomato (SMT), and Piccadilly tomato (PT). The histograms represent mean ± SD. Results were analysed by one-way analysis of variance (ANOVA) followed by Holm-Sidak’s multiple comparisons test. Different letters indicate significant differences within each fraction among different tomato cultivars (*p* < 0.001).

In detail, regarding the peel, the highest levels of total water-soluble and fat-soluble antioxidant capacity were measured in Cherry, while the lowest were found in Smooth round tomato, Round sauce, and Piccadilly. In the seeds, the highest values of both antioxidant activities were observed in Cherry and the lowest in Piccadilly. Finally, concerning the pulp, Cherry and Datterino showed the highest water-soluble and fat-soluble activities, while the lowest levels were in Piccadilly (water-soluble antioxidant capacity), and San Marzano and Piccadilly (fat-soluble antioxidant capacity) (Figure 1a,b, respectively).

The multiple comparisons among the three fractions showed a significant difference (*p* < 0.001) between both water-soluble and fat-soluble antioxidant capacities measured in peel vs. seeds, peel vs. pulp, and seeds vs. pulp.

**Figure 2:**
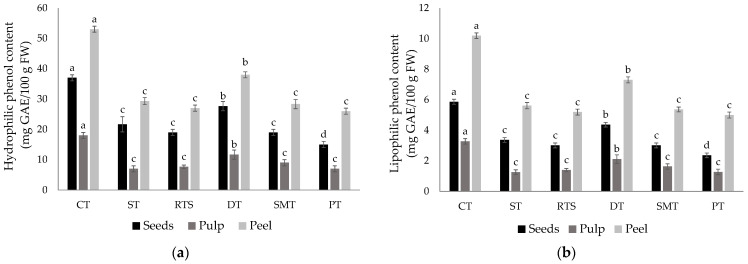
Hydrophilic (**a**) and lipophilic phenol content (**b**) in the seeds, pulp, and peel fractions of each tomato cultivar: Cherry tomato (CT), Smooth round tomato (ST), Round tomato sauce (RTS), Datterino tomato (DT), S. Marzano tomato (SMT), and Piccadilly tomato (PT). The histograms represent mean ± SD. Results were analysed by one-way analysis of variance (ANOVA) followed by Holm-Sidak’s multiple comparisons test. Different letters indicate significant differences within each fraction among different tomato cultivars (*p* < 0.001).

The highest content of hydrophilic and lipophilic phenols was found in the peel compared to both seeds and pulp. Moreover, within each variety, the phenolic content in the seeds was always higher than in the pulp (Figure 2a,b, respectively).

In particular, in the peel, the highest content of hydrophilic and lipophilic phenols was found in Cherry, while the lowest was found in Smooth round, Round sauce, San Marzano, and Piccadilly tomatoes. Comparing the seeds of all cultivars, the highest and lowest levels of phenols were measured in Cherry and Piccadilly, respectively. Furthermore, the highest phenolic content was determined in the Cherry pulp, while the lowest was in all other cultivars except Datterino (Figure 2a,b).

The content of hydrophilic and lipophilic phenols in the three fractions of each cultivar was always statistically different (*p* < 0.001).

**Figure 3:**
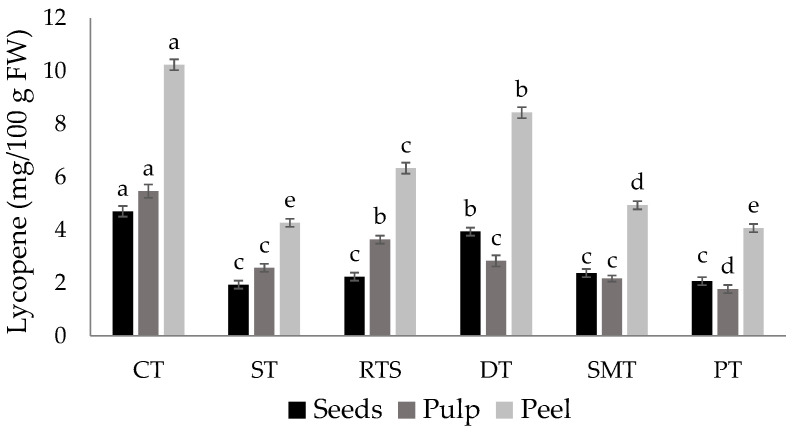
Lycopene content in the seeds, pulp, and peel fractions of each tomato cultivar: Cherry tomato (CT), Smooth round tomato (ST), Round tomato sauce (RTS), Datterino tomato (DT), S. Marzano tomato (SMT), and Piccadilly tomato (PT). The histograms represent mean ± SD. Results were analysed by one-way analysis of variance (ANOVA) followed by Holm-Sidak’s multiple comparisons test. Different letters indicate significant differences within each fraction among different tomato cultivars (*p* < 0.001).

All cultivars showed the highest lycopene concentration in the peel compared to the seeds and pulp. Lycopene levels were higher in the seeds than in the pulp of Datterino (*p* < 0.001), S. Marzano (*p* < 0.05), and Piccadilly (*p* < 0.01) varieties. Conversely, in the other cultivars, lycopene was more concentrated in the pulp fraction. In detail, in the seeds, the highest concentration of lycopene was found in Cherry tomato and the lowest in all the other cultivars except Datterino tomato. In the pulp, the highest content was measured in Cherry tomato and the lowest in Piccadilly. Finally, in the peel, the highest concentration of lycopene was found in Cherry tomato and the lowest in Smooth round and Piccadilly tomatoes (Figure 3).

The *p*-values in Table 2 indicate a significant difference between the lycopene content measured in seeds vs. pulp, seeds vs. peel, and pulp vs. peel because they are always lower than 0.05.

Finally, regardless of the cultivar, the peel showed the highest ascorbic acid content compared to the seeds and pulp fractions, while no significant differences were observed between seeds and pulp.

**Figure 4:**
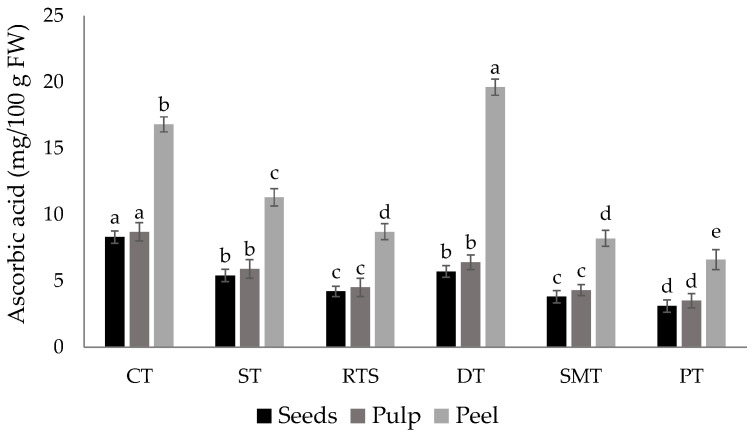
Ascorbic acid content in the seeds, pulp, and peel fractions of each tomato cultivar: Cherry tomato (CT), Smooth round tomato (ST), Round tomato sauce (RTS), Datterino tomato (DT), S. Marzano tomato (SMT), and Piccadilly tomato (PT). The histograms represent mean ± SD. Results were analysed by one-way analysis of variance (ANOVA) followed by Holm-Sidak’s multiple comparisons test. Different letters indicate significant differences within each fraction among different tomato cultivars (*p* < 0.001).

In the peel, the highest concentration of ascorbic acid was measured in Datterino and the lowest in Piccadilly tomatoes. In seeds and pulp, the highest content was in Cherry tomato and the lowest in Piccadilly (Figure 4).

No significant differences were observed among the three fractions of each tomato cultivar (Table 3).

### 3.2. Antioxidant Capacity and Content in Tomato Purees

The total antioxidant capacity of fresh and pureed tomatoes was determined by ABTS and DPPH assays. The total antioxidant capacity levels were consistently higher in the purees than in the fresh tomatoes with both methods. Furthermore, the measured values showed no significant differences (Table S1). Finally, total phenolic and lycopene content in tomato purees was also higher than those measured in whole fresh tomatoes of each cultivar. On the contrary, ascorbic acid concentration was always lower (Table 4).

In detail, the maximum phenolic (1.4 times) and lycopene (12.7 times) content were found in Cherry tomato, while the lowest were found in Piccadilly (1.24 times total phenolic content and 6.36 times lycopene content). The highest decrease of ascorbic acid concentration was determined in Cherry tomato (5.00 times), while the lowest (3.33 times) was determined in Piccadilly.

### 3.3. Percent Contribution of the Peel, Pulp, and Seeds Fractions to the Total Antioxidant Content in Whole Tomatoes

The results referring to 100 g FW of each tomato evidenced that antioxidant content and capacities were always higher in the peel than in the pulp and seeds. As the quantification of the peel and seeds fractions present in a whole fresh tomato was lower than the pulp (Table 1), it was necessary to determine the amount of antioxidants in each fraction based on their actual weights. Thus, the percentage contribution of the peel, pulp, and seeds fractions to the total antioxidant content in whole tomatoes was calculated, and the results were reported in Table 5.

The lowest contribution (51%) of the peel and seeds fractions to total antioxidant capacity was found in Datterino tomatoes, while the highest (63%) was found in S. Marzano and Piccadilly tomatoes. Conversely, the most significant contribution to the total phenolic content was observed in the Smooth round tomato (65%), while the lowest (56%) was found in the Cherry tomato. Furthermore, the peel and seeds provided the lowest contribution (38%) to lycopene in the Smooth round tomato and Round tomato sauce*,* and the highest (51%) in Datterino. Finally, the less consistent contribution of the peel and seed fractions to ascorbic acid was found in the Cherry tomato (41%), while the highest contribution was observed in the cultivar Datterino (47%).

## 4. Discussion

Fruits and vegetables are rich in bioactive compounds beneficial for human health. However, they are not always consumed entirely and they are often subjected to processing to separate the valuable product from other plant constituents [41]. It has been estimated that many vegetables and fruits produce 25–30% of inedible products [42], but the inability or impossibility to recover waste materials such as peels, seeds, and stones determines a considerable loss of antioxidants from natural sources [43].

In particular, the peel is widely reported to be richer in antioxidants than other fruit components. For instance, phenolic compounds and ascorbic acid are more concentrated in the peel than in the pulp of citrus fruits [44]. The phenolic content of the edible pulp of bananas (*Musa paradisiaca*) is about 25% of that in the peel [45]. The peel and other residues and by-products of star fruit (*Averrhoa carambola* L.), pomegranate (*Citrus paradise* M., *Punica granatum* L.), banana (*Musa acuminata Colla*), and citrus are evaluated as relevant sources of antioxidants [46,47,48,49,50]. Moreover, Wolfe, Wu, and Liu [51] demonstrated that phenol concentration, antioxidant activity, and antiproliferative activity measured in apple peel are significantly higher than in the pulp. For this reason, they hypothesize that daily consumption of apple peels reduces the risk of cardiovascular disease and cancer.

In addition to the concentration of antioxidants, the antioxidant activity of many fruits (guava, kiwifruit, purple mulberry, strawberry, white pomegranate) is also higher in the peel and seed fractions than in the pulp [52].

In the past, great attention was paid to the antioxidant content in tomatoes, which represent the main component of the Mediterranean diet. Consuming fresh tomato berries or tomato puree offers significant health benefits, as they are rich in antioxidant compounds essential in preventing various diseases associated with oxidative stress [53,54,55,56].

The lycopene, phenols, flavonoids, ascorbic acid, and vitamin E in tomatoes are mainly responsible for the antioxidant capacity due to their ability to quench free radicals, which are responsible for oxidative changes in the human body [57,58,59].

Several studies have already shown that the peel and seeds are often removed in the daily consumption of tomatoes and the preparation of their derivatives, despite being a valuable source of bioactive compounds and minerals [12,27,60,61].

More recently, a study on 12 field-grown tomato genotypes reported that, on average, lycopene levels in the tomato peel are 2.5 times higher than in the pulp [62]. The same authors also noted that the tomato peel contains many phenols and ascorbic acid.

Significant differences in the antioxidant content of the seeds, pulp, and peel fractions have been measured in different Indian tomato cultivars developed at high altitudes and in the lowlands. In all the tested cultivars, the highest antioxidant levels and free radical scavenging activities were found in the peel. In detail, the highest lycopene content was found in high-altitude cultivars, and the highest ascorbic acid and phenol levels were detected in plain region cultivars [63].

Based on this knowledge, our study evaluated the effect of peel and seeds removal on ascorbic acid, phenol, and lycopene content and antioxidant capacity in tomato fruits of six typical varieties cultivated in Campania, a region in Southern Italy.

Data reported in the literature (on 100 g of analysed fractions) indicate that the peel is the tomato fruit component with the highest concentration of antioxidants, namely water-soluble and fat-soluble antioxidant capacity, phenolics, lycopene, and ascorbic acid, followed by the seeds and pulp. Furthermore, the antioxidant content measured in the seeds and peel, and calculated taking into account the tomato weights (Table 5), confirms the considerable contribution of these components to the scavenging properties of tomato fruits (i.e., antioxidant capacity from 51 to 63%; total phenolic content from 56% to 65%; lycopene content from 38% to 51%; ascorbic acid content from 41% to 47%). Since for all studied tomato cultivars the peel represents a precious sink of bioactive compounds, it is noteworthy that peeling is the most detrimental procedure before consuming tomatoes, especially for Cherry and Datterino varieties, which are considered the healthiest cultivars.

It is well known that domestic and industrial food processing significantly affects the structural integrity of fruits and vegetables [64]. In particular, industrial processing often involves many thermal processes, which may positively or negatively impact food. More specifically, they can inactivate food-borne enzymes and pathogens, increase foodstuff’s digestibility and bioavailability, extend the shelf life of fruits, or lead to the loss of some desirable nutrients [65].

Several factors have already been demonstrated to influence the capacity and content of antioxidants during industrial tomato processing [31,66,67,68]. However, to date, no information is available on the effects of processing during the production of homemade tomato puree, a common practice in Campania (Southern Italy) in the summer, from July to September.

Therefore, we evaluated for the first time whether and to what extent the antioxidant capacity and the content of phenols, lycopene, and vitamin C could change in homemade tomato puree compared to unprocessed tomatoes. Although San Marzano is usually the most commonly used for homemade passata, we prepared purees with the six available tomato cultivars.

Our data show that home processing positively affects phenols and lycopene content. We hypothesize that heating glass jars filled with tomato puree at 100 °C for 40 min could have promoted the extractability and release of (bound) phenols [31] and lycopene from tissues. The heating process could have also: induced lycopene isomerization from all-trans to cis configuration, increased its bioavailability [69,70,71], and deactivated endogenous oxidative enzymes responsible for the degradation of antioxidant compounds [72].

In addition, the mechanical treatment (sieving) during tomato processing could have favoured phenolics’ bioaccessibility, extractability, and bioavailability [72].

Finally, the evidence that total antioxidant capacity is always higher in purees than in fresh tomatoes suggests that the increased phenols and lycopene phenols compensates for the significant reduction in ascorbic acid (around 70%) (Table S1 and Table 4), probably due to oxidation processes [73]. Furthermore, in puree, the formation of polymeric phenols, which are more potent antioxidants than their simple counterparts [66], could be a further reason to explain the increase in antioxidant activity.

## 5. Conclusions

This study focused on the antioxidant characterization of six Italian tomato cultivars and evaluated the antioxidant content of homemade puree for the first time.

The first analysis highlights that consuming fresh and unpeeled berries is preferable because removing the peel and seed fractions significantly reduces the content of antioxidants and total antioxidant activities.

The second finding concerns the antioxidant properties of tomato purees. To date, the only data available in the literature refer to industrial purees. As a novel aspect, we have proven, for the first time, that homemade tomato purees, because of their high antioxidant content which is not lost during the preparation procedure, are strongly recommended in the human diet. All cultivars, especially Cherry tomatoes, provide purees rich in antioxidant capacity, and lycopene and phenol content. Such compounds, which become more available following the heating process, represent an added value in the diet, effectively preventing oxidative stress.

As a final consideration, our study provides encouraging results that valorise local products and support the consumption and reutilization of waste products in view of the circular economy designed to benefit the environment.

## Figures and Tables

**Table 1:** Weight of the seed, pulp, and peel fractions of six cultivars of tomatoes (means ± standard deviation, *n* = 3).

Cultivar/Fraction	Weight (g)	Cultivar/Fraction	Weight (g)
*Cherry tomato*		*Datterino*	
Seeds	4.1 ± 0.06	Seeds	2.9 ± 0.04
Pulp	11.7 ± 0.16	Pulp	10.1 ± 0.77
Peel	2.2 ± 0.03	Peel	2.1 ± 0.03
Total	18 ± 1.1	Total	15 ± 0.9
*Smooth round tomato*		*S. Marzano*	
Seeds	41.4 ± 1.96	Seeds	27.3 ± 1.12
Pulp	151.8 ± 5.28	Pulp	81.9 ± 2.92
Peel	36.8 ± 1.51	Peel	20.8 ± 1.08
Total	230 ± 7.6	Total	130 ± 4.6
*Round tomato sauce*		*Piccadilly*	
Seeds	20.9 ± 1.09	Seeds	6.1 ± 0.09
Pulp	71.5 ± 2.73	Pulp	20.5 ± 1.04
Peel	17.6 ± 0.87	Peel	5.4 ± 0.08
Total	110 ± 4.0	Total	32 ± 1.3

**Table 2:** Comparison among lycopene levels in the seeds, pulp, and peel fractions of each cultivar.

Cultivar/Fraction	Lycopene	Cultivar/Fraction	Lycopene
	*p*-Value		*p*-Value
*Cherry tomato*		*Datterino tomato*	
Seeds vs. Pulp	**<0.001**	Seeds vs. Pulp	**<0.001**
Seeds vs. Peel	**<0.001**	Seeds vs. Peel	**<0.001**
Pulp vs. Peel	**<0.001**	Pulp vs. Peel	**<0.001**
*Smooth round tomato*		*S. Marzano tomato*	
Seeds vs. Pulp	**<0.001**	Seeds vs. Pulp	**0.04**
Seeds vs. Peel	**<0.001**	Seeds vs. Peel	**<0.001**
Pulp vs. Peel	**<0.001**	Pulp vs. Peel	**<0.001**
*Round tomato sauce*		*Piccadilly tomato*	
Seeds vs. Pulp	**<0.001**	Seeds vs. Pulp	**0.008**
Seeds vs. Peel	**<0.001**	Seeds vs. Peel	**<0.001**
Pulp vs. Peel	**<0.001**	Pulp vs. Peel	**<0.001**

**Table 3:** Comparison of lycopene levels among the seeds, pulp, and peel fractions of each cultivar.

Cultivar/Fraction	Ascorbic Acid	Cultivar/Fraction	Ascorbic Acid
	*p*-Value		*p*-Value
*Cherry tomato*		*Datterino tomato*	
Seeds vs. Pulp	0.26	Seeds vs. Pulp	0.14
Seeds vs. Peel	**<0.001**	Seeds vs. Peel	**<0.001**
Pulp vs. Peel	**<0.001**	Pulp vs. Peel	**<0.001**
*Smooth round tomato*		*S. Marzano tomato*	
Seeds vs. Pulp	0.12	Seeds vs. Pulp	0.14
Seeds vs. Peel	**<0.001**	Seeds vs. Peel	**<0.001**
Pulp vs. Peel	**<0.001**	Pulp vs. Peel	**<0.001**
*Round tomato sauce*		*Piccadilly tomato*	
Seeds vs. Pulp	0.26	Seeds vs. Pulp	0.27
Seeds vs. Peel	**<0.001**	Seeds vs. Peel	**<0.001**
Pulp vs. Peel	**<0.001**	Pulp vs. Peel	**<0.001**

**Table 4:** Total phenolic content, lycopene, and ascorbic acid content in tomato purees.

Cultivar	Total Phenolic Content (Hydrophilic + Lipophilic) (mg GAE/100 g)		Lycopene (mg/100 g)		Ascorbic Acid (mg/100 g)	
	Fresh Tomato	Puree	Fresh Tomato	Puree	Fresh Tomato	Puree
*Cherry tomato*	31.3 ± 1.08	43.8 ± 1.33	5.9 ± 0.20	75.0 ± 2.00	9.6 ± 0.46	1.9 ± 0.07
*Smooth round tomato*	15.5 ± 0.94	20.4 ± 1.08	2.7 ± 0.20	21.6 ± 1.18	6.7 ± 0.45	1.5 ± 0.05
*Round tomato sauce*	15.2 ± 0.83	19.2 ± 1.06	3.3 ± 0.21	23.3 ± 1.44	5.1 ± 0.31	1.3 ± 0.04
*Datterino tomato*	21.7 ± 0.96	29.7 ± 1.21	3.8 ± 0.20	38.9 ± 1.15	8.1 ± 0.45	1.7 ± 0.06
*S. Marzano tomato*	16.7 ± 0.85	22.2 ± 1.13	2.7 ± 0.15	25.2 ± 1.05	4.8 ± 0.46	1.3 ± 0.05
*Piccadilly tomato*	13.9 ± 0.82	17.2 ± 1.04	2.2 ± 0.20	14.0 ± 1.00	4.0 ± 0.31	1.2 ± 0.03

**Table 5:** Percentage contributions of the seeds, pulp, and peel fractions to the total antioxidant content.

Cultivar/Fraction	Antioxidant Capacity	Total Phenolic Content	Lycopene	Ascorbic Acid
	(Soluble + Fat-Soluble)	(Hydrophilic + Lipophilic)		
*Cherry tomato*				
Seeds	31	32	18	20
Pulp	43	44	61	59
Peel	25	24	21	21
*Smooth round tomato*				
Seeds	25	29	13	15
Pulp	43	35	62	58
Peel	32	36	25	27
*Round tomato sauce*				
Seeds	24	27	11	16
Pulp	44	39	62	57
Peel	33	34	27	27
*Datterino tomato*				
Seeds	23	28	20	13
Pulp	50	43	50	53
Peel	28	29	31	34
*S. Marzano tomato*				
Seeds	27	28	19	17
Pulp	37	40	51	56
Peel	36	32	30	27
*Piccadilly tomato*				
Seeds	24	24	18	15
Pulp	37	38	51	57
Peel	39	38	31	28

## Data Availability

Data is available from the corresponding authors upon reasonable request.

## Supplementary Materials

The following supporting information can be downloaded at: https://www.mdpi.com/article/10.3390/antiox12030761/s1, Table S1: Antioxidant capacity by ABTS and DPPH assays in whole fresh tomato and puree.
