# Supplementary material for: Antioxidant Characterization of Six Tomato Cultivars and Derived Products Destined for Human Consumption
Source: Antioxidants (Basel). 2023 Mar 21;12(3):761. doi: 10.3390/antiox12030761 (PMC10045220; doi:10.3390/antiox12030761)
Supplement: Supplementary file 1 [file antioxidants-12-00761-s001.zip › antioxidants-2280835-supplementary.pdf]

Table S1. Antioxidant capacity by ABTS and DPPH assays in whole fresh tomato and puree.

**Cultivar**	**ABTS**		**DPPH**	
	**Fresh tomato** (soluble + fat-soluble) (μM/100 g)	**Puree** (μM/100 g)	**Fresh tomato** (soluble + fat-soluble) (μM/100 g)	**Puree** (μM/100 g)
Cherry tomato	269 ± 7.2[a]	323 ± 7.5[b]	260 ± 12.3[a]	318 ± 7.6[b]
Smooth round tomato	188 ± 6.9[a]	242 ± 7.1[b]	181 ± 8.9[a]	236 ± 8.1[b]
Round tomato sauce	187 ± 6.8[a]	238 ± 7.0[b]	179 ± 10.0[a]	232 ± 6.7[b]
Datterino tomato	221 ± 7.5[a]	274 ± 8.3[b]	216 ± 7.0[a]	268 ± 6.2[b]
S. Marzano tomato	181 ± 6.7[a]	228 ± 6.8[b]	174 ± 8.3[a]	221 ± 8.7[b]
Piccadilly tomato	154 ± 6.1[a]	201 ± 6.3[b]	148 ± 7.8[a]	196 ± 6.0[b]

Values represent mean ± standard deviation. Results were analyzed by one-way analysis of variance ANOVA followed by Holm-Sidak's multiple comparisons test. Data followed by similar letters in the same line are not significantly different for $p < 0.05$.
